# The mental health effects of changing from insecure to secure visas for refugees

**DOI:** 10.1177/00048674231177950

**Published:** 2023-05-29

**Authors:** Angela Nickerson, Yulisha Byrow, Meaghan O’Donnell, Richard A Bryant, Vicki Mau, Tadgh McMahon, Joel Hoffman, Natalie Mastrogiovanni, Philippa Specker, Belinda J Liddell

**Affiliations:** 1 School of Psychology, University of New South Wales, Sydney, NSW, Australia; 2 Phoenix Australia Centre for Posttraumatic Mental Health, Carlton, Victoria, Australia; 3 Department of Psychiatry, Faculty of Medicine, Dentistry, and Health Sciences, University of Melbourne, Parkville VIC, Australia; 4 Australian Red Cross, North Melbourne, VIC, Australia; 5 SSI, Ashfield, NSW, Australia; 6 College of Public Health and Medicine, Flinders University, Adelaide, SA, Australia

**Keywords:** Refugees, asylum-seekers, depression, social functioning

## Abstract

**Objective:**

In response to growing numbers of refugees worldwide, host governments are increasingly implementing temporary protection policies; however, little is known regarding the mental health impact of these policies. This online longitudinal study investigated whether refugees who transitioned from low visa security (e.g. short-term transient visas) to medium (e.g. temporary protection visas) or high visa (e.g. permanent visas) security showed changes in depression symptoms, social difficulties and immigration-related fears.

**Methods:**

Participants were 1,201 refugees and asylum-seekers from Arabic, Farsi, Tamil or English-speaking backgrounds. Study variables were measured prior to and after change in visa status (6 months apart).

**Results:**

Refugees who transitioned from low to medium security visas showed reduced immigration-related fear (*B* = −0.09, 95% confidence interval = −0.29 to −0.06), but no change in depression symptoms or social difficulties compared to those who retained low visa security. Refugees who transitioned from low to high security visas showed reduced depression symptoms (*B* = −0.02, 95% confidence interval = −0.04 to −0.01), social difficulties (*B* = −0.04, 95% confidence interval = −0.05 to −0.01) and immigration-related fear (*B* = −0.03, 95% confidence interval = −0.06 to −0.01) compared to those who retained low visa security.

**Conclusion:**

Findings indicate that the increased security afforded by temporary protection policies (vs short-term transient visas) did not translate into improved mental health and social outcomes for refugees. In contrast, permanent protection was associated with significant improvements in psychological and social functioning. These results have important policy implications for countries who have committed to protect and facilitate improved mental health among refugees.

More than 100 million people have been forcibly displaced by war and persecution worldwide (Bevan and Higgins, 2002; UNHCR, 2023). The number of refugees and asylum-seekers seeking protection far exceeds the number of available resettlement places in high income host-countries, with less than 1% of forcibly displaced people being permanently resettled in 2021 (UNHCR, 2022). Consequently, refugees around the world live in various states of insecurity, ranging from low levels to high levels of security. The majority of refugees live in a state of low security without a permanent visa (e.g. asylum seeker with no visa, a form of bridging visa), with reduced rights and restricted access to employment, financial support, education or healthcare (Al-Rousan et al., 2018; UNHCR, 2009). Furthermore, there has been a global movement towards temporary protection visas as an alternative to increasing permanent resettlement places in countries who are signatories to international agreements for the protection of refugees (Andrew & Renata Kaldor Centre for International Refugee Law, 2000b).

Temporary protection visas offer a medium level of security: visa holders are recognized as legitimate refugees, can typically access basic health, financial, education and social services, but are required to periodically re-apply for protection. For example, until recently in Australia, refugees on a temporary protection visa (TPV) or a safe haven enterprise visa (SHEV) were required to re-apply for their visa every 3 or 5 years respectively, and had no access to family reunion pathways (Law, 2022b). This temporary status meant that individuals lived in a constant state of uncertainty over their future, which was exacerbated by the risk of a negative decision upon reapplication and possible deportation.^1^ It is not known, however, whether the medium level of security afforded by temporary protection visas translates into better mental health outcomes for refugees compared to low security visas.

Cross-sectional and longitudinal research has demonstrated that refugees who have or maintain temporary protection visa status show poorer mental health outcomes compared to those with permanent visas (Momartin et al., 2006; Newnham et al., 2019; Nickerson et al., 2019; Rostami et al., 2022; Steel et al., 2006, 2011). Furthermore, one longitudinal study has found that mental health improves with the conversion of temporary into permanent visa status (Nickerson et al., 2011). No study to date, however, has investigated the relative psychological impact of medium-security temporary protection visas compared to low-security short-term visas; nor the mental health consequences of transitioning to temporary protection visas (compared to permanent visas) from other short-term visas. Moreover, there has been little consideration of the impact of different visas on settlement stressors such as social integration (e.g. feeling isolated) or immigration-related (e.g. fear of deportation) difficulties. This leaves a substantial knowledge gap regarding the mental health consequences of temporary protection visas. This is of critical importance as the provision of an environment supporting positive mental health and social functioning is a central component of international protection policies (UNHCR, 2021).

To address this question, we assessed whether *changes* in visa security were associated with differential patterns of depression symptoms, immigration-related fear and social difficulties in a large cohort of refugees living in Australia. Participants in this study comprised those who (1) maintained low visa security, (2) changed from low to medium visa security, (3) changed from low to high visa security, (4) maintained medium visa security or (5) maintained high visa security. If temporary protection policies do indeed afford refugees with a sense of security, we would expect that moving from low to medium visa security would be associated with significant decreases in depression symptoms, immigration-related fears and social integration difficulties, compared to refugees who maintain low security visa status.

## Method

### Participants and procedure

Participants were 1,021 Arabic, Farsi, Tamil or English-speaking refugees or asylum-seekers living in Australia. Participants had taken part in a longitudinal study investigating refugee mental health over eight time-points from 2015 to 2020 (Liddell et al., 2021; Nickerson et al., 2018, 2019). Study languages were selected as they represented over 50% of successful applications for asylum in Australia between 2012 and 2015 (Australian Government Department of Immigration and Border Protection, 2013, 2014a, 2014b). Participants were recruited via advertisements at refugee support services across Australia, social media platforms (e.g. Facebook) and snowball sampling, which has been found to be effective for difficult-to-access populations such as refugees (Sadler et al., 2010).

Data collection for time-point 1 of the Refugee Adjustment Study was undertaken between April 2015 and January 2018. Time-points 2 to 5 were collected at 6-month intervals, and time-points 6 to 8 were collected at 12-month intervals. Participants registered their interest in participating on the study website, were screened to assess eligibility (refugee or asylum-seeking background living in the Australian community, arrival in Australia in or after January 2011, aged 18+, able to read in Arabic, Farsi, Tamil or English), and eligible participants were sent a personalized link to the study, which they completed online via the KeySurvey platform. Each survey took ~45 minutes to complete. Participants without Internet access completed paper versions of the survey and returned these by post. Six months after the first time-point, participants were sent a link to or hard copy of the second survey, and this procedure was repeated for subsequent time-points. An $AUD25 shopping voucher was provided at each time-point. The authors assert that all procedures contributing to this work comply with the ethical standards of the relevant national and institutional committees on human experimentation and with the Helsinki Declaration of 1975, as revised in 2008. All procedures involving human subjects/patients were approved by the UNSW Human Research Ethics Committee (HC14106 and HC180627).

Participants were eligible for inclusion in the current study if they had data available regarding visa status for at least two out of the eight time-points in the study and fell into one of the following groups: maintained high visa security (*n* = 820, 82.1%, e.g. permanent protection visa and Australian citizen) across all time-points, maintained middle visa security (*n* = 22, 2.2%, e.g. temporary protection visa) across all time-points, maintained low visa security (*n* = 109, 10.9%, e.g. bridging visa and expired visa) across all time-points, changed from low to medium visa security (*n* = 54, 5.4%, e.g. temporary protection visas), or changed from low to high visa security (*n* = 16, 1.6%). The number of participants in the Refugee Adjustment Study who changed from medium to high visa security (*n* = 3, 0.01%) and from middle to low visa security (*n* = 7, 0.01%) were inadequate to include in analyses, which reflected Australia’s policy at the time of data collection (Law, 2022b).

#### Measures

All measures were translated and blind back-translated into Arabic, Farsi and Tamil by accredited interpreters, with discrepancies being reconciled by the research team and interpreters experienced in working with mental health constructs. We then pilot-tested measures with individuals from a refugee background with a range of education levels from each language group, to ensure ease of understanding irrespective of education level.

*Demographic* information including age, gender, country of origin, mode of travel to Australia (airplane or boat) and date of arrival in Australia was included in this study.

*Exposure to potentially traumatic events (PTEs)* was measured at time-point 1 using the 16-item Harvard Trauma Questionnaire (Mollica et al., 1992), which indexes traumatic events commonly experienced by refugees. We derived a total count of diversity of PTE exposure, which represented the number of types of PTE events the individual had experienced and/or witnessed, ranging from 0 to 16.

*Social integration difficulties* were measured using three items from the 25-item adapted version of the Post-Migration Living Difficulties Checklist (Steel et al., 1999). Items used in this study comprised *‘loneliness’, ‘isolation’* and *‘boredom’.* Each living difficulty was rated on a five-point scale (1 = *was not a problem/did not happen, 5* = *a very serious problem*). This scale showed strong internal consistency (e.g. α = 0.91 at time-point 1).

*Immigration-related fears* were measured using four items from the adapted Post-Migration Living Difficulties Checklist (Steel et al., 1999). These items comprised ‘fear of being sent home’, ‘fear of being sent to an offshore processing centre’, ‘fear your visa status will never be resolved’ and ‘difficulties with immigration officials’. This scale showed strong internal consistency (e.g. α = 0.81 at time-point 1).

*Depression symptoms* were measured using the nine-item Patient Health Questionnaire (Kroenke et al., 2001). Participants indicated on a four-point scale (0 = *not at all*, 3 = *nearly every day*) how often they had been bothered by symptoms in the past 2 weeks. This scale showed strong internal consistency (e.g. α = 0.93 at time-point 1).

#### Data preparation

To identify participants who were appropriate for the current analysis, we first extracted visa status information from the longitudinal dataset for all participants at all time-points, and coded this into low, medium and high visa security groups. If a participant changed visa security group at some point during this study (i.e. over the 6 year time period spanning April 2015–March 2020), this change was verified using visa information provided at surrounding time-points to ensure accuracy. If necessary, information was clarified with participants who had previously given permission to be contacted via telephone. The time-point prior to change in visa security group and the time-points after change in visa security group were then extracted for these participants. For participants who did not change visa status (i.e. those who remained in the low and high visa security groups), all available time-points were included in the analysis. We verified that there was no change in visa status across subsequent time-points, and for one participant where this could not be verified, coded data as missing for time-points that not were verifiable. As participation in this study was not temporally linked to a specific event (e.g. displacement), we controlled for time in Australia in analyses to ensure findings were not accounted for by length of time in the host country. We determined approximate time in Australia prior to visa change by calculating the difference in years between date of arrival in Australia and date of completion of the time-point representing visa security group change.

### Data analysis

We first examined differences between visa change groups on participant characteristics including age, gender, country of origin, mode of travel to Australia, time in Australia before visa change and PTE exposure. Next, linear mixed models analyses were used to investigate whether change in depression symptoms, immigration-related fear and social difficulties differed according to change in visa status over time. Linear mixed models allowed us to investigate whether changes in depression symptoms, immigration fear and social difficulties showed different patterns of change over time for different visa status change groups. All available data points were included for each participant: the data point prior to visa change (baseline), the data point immediately after visa change and the subsequent data points. Linear mixed models is a flexible statistical technique that allows for modelling of between-person differences as well as within-person change while using all available data (Raudenbush and Bryk, 2001). Specifically, linear mixed models allows for the estimation of a slope where there are two or more data points. In the model, we included a variable representing time where the time-point prior to visa change was coded as 0, and each subsequent time-point was coded according to the number of months after the first time-point (the time-point prior to visa change) that the participant completed the survey. Also included in the model were dummy-coded variables representing visa change group (remain high visa security, remain medium visa security, change low to medium visa security, change low to high visa security; remain low visa security represented the reference group). Interaction variables were included in the model to allow us to investigate differential change in outcomes over time for visa change groups. These were represented by interactions between time and visa change for each dummy-coded visa change group (e.g. time × remain high visa security). Accordingly, change over time for each visa change group was evaluated in reference to those who maintained low visa security over time. This allowed us to determine whether mental health and social outcomes improved for those who moved from low to medium security, from low to high security, or retained medium or high security compared to those who retained low visa security. We included covariates in the model, namely, age, gender, PTE exposure and time in Australia prior to visa change. Country of origin and mode of travel to Australia were not included in covariates as they mapped directly on visa security groups (see Table 1). Random intercepts were included in all models. We investigated whether the inclusion of random slopes improved the fit of all models, using a loglikelihood difference test. Results were considered significant when *p* < 0.05. All analyses were undertaken in SPSS version 28 and used an unstructured covariance structure.

**Table 1.** Participant characteristics.

	Overall sample *n* = 1021	Maintain high visa security *n* = 820	Maintain medium visa security *n* = 22	Maintain low visa security *n* = 109	Change low to medium visa security *n* = 54	Change low to high visa security *n* = 16	
Age	38.42 (11.96)	39.21 (12.50)^a^	35.20 (10.44)	34.88 (8.35)^b^	35.35 (8.49)	38.10 (11.62)	*F*(985) = 4.54, *p* < 0.001
Gender (male)	551 (56.0%)	416 (53.0%)	19 (86.4%)	77 (72.0%)	31 (57.4%)	8 (50.0%)	χ^2^(3) = 22.45, *p* ⩽ 0.001
Country of origin							χ^2^(15) = 729.19, *p* < 0.001
Iraq	575 (56.3%)	559 (68.2%)	1 (4.5%)	7 (6.4%)	3 (5.6%)	5 (31.3%)	
Syria	167 (16.4%)	166 (20.2%)	0 (0.0%)	0 (0.0%)	0 (0.01%)	1 (6.3%)	
Iran	147 (14.4%)	44 (5.4%)	10 (45.5%)	50 (45.9%)	41 (75.9%)	2 (12.5%)	
Afghanistan	33 (3.2%)	24 (2.9%)	1 (4.5%)	6 (5.5%)	2 (3.7%)	0 (0.0%)	
Sri Lanka	42 (4.1%)	2 (0.2%)	10 (45.5%)	24 (22.0%)	5 (9.3%)	1 (6.3%)	
Other (e.g. Burma, Pakistan, and Somalia)	57 (5.6%)	25 (3.0%)	0 (0.0%)	22 (20.2%)	3 (5.6%)	7 (43.8%)	
Mode of travel							
Plane	832 (81.5%)	792 (96.6%)	1 (4.5%)	23 (21.1%)	1 (1.9%)	15 (93.8%)	χ^2^(3) = 662.23, *p* < 0.001
Boat	189 (18.5%)	28 (3.4%)	21 (95.5%)	86 (78.9%)	53 (98.1%)	1 (6.3%)	
Time in Australia (before visa change)	2.93 (1.67)	2.39 (1.31)^a,c,d,e^	5.58 (0.94)^b,e^	4.80 (1.18)^b,d^	5.63 (0.91)^a,b,e^	4.11 (1.89)^a,c,d^	*F*(980) = 173.59, *p* < 0.001
PTE exposure	4.11 (4.38)	3.26 (3.82)^a,c,d,e^	8.81 (4.74)^b^	7.47 (4.91)^b^	7.48 (4.48)^b^	5.69 (4.94)^b^	*F*(981) = 44.75, *p* < 0.001
Depression symptoms (time-point prior to visa change)	0.89 (0.75)	0.78 (0.69)^a,c,d,e^	1.43 (0.78)^b^	1.33 (0.81)^b^	1.37 (0.84)^b^	1.40 (0.22)^b^	*F*(977) = 24.13, *p* < 0.001
Immigration-related fear (time-point prior to visa change)	1.83 (1.09)	1.46 (0.70)^a,c,d,e^	3.33 (0.88)^b^	3.57 (0.93)^b,e^	3.74 (0.95)^b,e^	2.52 (1.33)^a,b,d^	*F*(871) = 246.01, *p* < 0.001
Social difficulties (time-point prior to visa change)	2.13 (1.24)	1.91 (1.12)^a,c,d,e^	3.27 (1.42)^b^	2.85 (1.31)^b^	3.39 (1.27)^b^	2.90 (1.43)^b^	*F*(987) = 36.87, *p* < 0.001

PTE: potentially traumatic event.

Coefficients significant at *p* < 0.01.

a Significantly different to maintain low visa security group.

b Significantly different to maintain high visa security group.

c Significantly different to maintain medium visa security group.

d Significantly different to change low to medium visa security group.

e Significantly different to change low to high visa security group.

## Results

### Participant characteristics

Participants who changed from low to medium visa security were most likely to be from Iran, to have travelled to Australia by boat, to have experienced a higher number of PTEs and to have been in Australia longer than other groups (Table 1). Participants who moved from low to high visa security were most likely to be from Iraq and to have travelled by plane.

### Baseline differences in depression, immigration-related fear and social difficulties

At baseline, participants who maintained high visa security reported lower depression symptoms, immigration-related fear and social difficulties than all other groups. At baseline, participants in the low to high visa security group showed lower immigration-related fears than those who remained in the low visa security group. At baseline, participants in the low to medium security visa group showed higher immigration-related fears and social difficulties than those who remained in the low visa security group.

### Linear mixed models

Investigation of models with a random intercept only compared to those with a random intercept and random slope indicated that the inclusion of a random slope improved the fit of all models (Depression: LLΔ (2) = 66.33, *p* < 0.001; Immigration-related fears LLΔ (2) = 40.27, *p* < 0.001, Social integration difficulties LLΔ (2) = 81.30, *p* < 0.001) (Table 2). This suggests that there was substantial variation in outcomes at time-point 1, and in change over time between participants. Accordingly, random intercepts and random slopes were modelled for all variables.

**Table 2.** Linear mixed models results.

	Depression				Immigration fear				Social difficulties			
	*B*	SE	*p*-value	95% CI	*B*	SE	*p*-value	95% CI	*B*	SE	*p*-value	95% CI
Intercept	0.64	0.11	<0.001	[0.43, 0.85]	2.76	0.12	<0.001	[2.52 to 3.01]	1.74	0.17	<0.001	[1.40, 2.06]
Gender (male)	−0.14	0.04	<0.001	[−0.21, −0.07]	0.04	0.04	0.392	[−0.05, 0.11]	−0.17	0.06	0.003	[−0.28, −0.06]
Age	0.01	0.01	0.438	[−0.01, 0.01]	−0.01	0.01	0.662	[−0.01, 0.01]	0.00	0.01	0.828	[−0.01, 0.01]
Length of stay in Australia before visa change	0.06	0.01	<0.001	[0.03, 0.09]	0.07	0.02	<0.001	[0.04, 0.10]	0.11	0.02	<0.001	[0.07, 0.15]
PTE exposure	0.06	0.01	<0.001	[0.06, 0.07]	0.05	0.01	<0.001	[0.04, 0.05]	0.10	0.01	<0.001	[0.09, 0.11]
Time (months)					0.01	0.04	0.138	[−0.01, 0.01]	0.01	0.01	0.048	[0.00, 0.02]
Remain low visa security	–	–	–	–	–	–	–	–	–	–	–	–
Remain medium visa security	0.05	0.15	0.719	[−0.24, 0.35]	−0.78	0.16	0.083	[−0.59, 0.04]	0.27	0.23	0.246	[−0.18, 0.71]
Remain high visa security	−0.17	0.08	0.026	[−0.31, 0.02]	−1.64	0.08	<0.001	[−1.80, −1.47]	−0.31	0.11	0.007	[−0.54, 0.09]
Change low to medium visa security	−0.06	0.11	0.605	[−0.28, 0.17]	−0.01	0.12	0.974	[−0.24, 0.24]	0.33	0.18	0.063	[−0.02, 0.67]
Change low to high visa security	0.11	0.17	0.534	[−0.23, 0.44]	0.99	0.18	<0.001	[−0.13, −0.64]	0.10	0.26	0.707	[−0.42, 0.61]
Remain low visa security × time												
Remain medium visa security × time	−0.01	0.01	0.880	[−0.02, 0.01]	0.01	0.01	0.284	[−0.01, 0.02]	−0.01	0.01	0.674	[−0.03, 0.02]
Remain high visa security × time	−0.05	0.02	0.011	[−0.01, −0.01]	−0.01	0.01	0.065	[−0.02, 0.00]	−0.01	0.01	0.008	[−0.02, −0.01]
Change low to medium visa security × time	0.01	0.01	0.975	[−0.04, 0.01]	−0.01	0.01	0.050	[−0.02, 0.00]	−0.01	0.01	0.525	[−0.02, 0.01]
Change low to high visa security × time	−0.02	0.01	0.014	[−0.04, −0.01]	−0.03	0.01	0.001	[−0.05, −0.01]	−0.04	0.01	0.004	[−0.06, −0.01]

SE: standard error; CI: confidence interval; PTE: potentially traumatic event.

**Figure 1.**
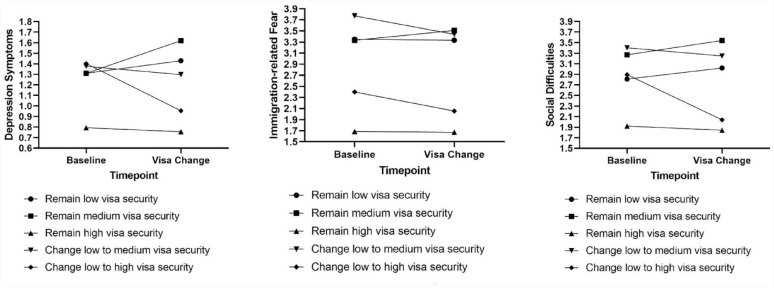
Changes in depression symptoms, social difficulties and immigration-related fear according to visa status change.

For depression symptoms, there was a significant interaction between time and change between the low to high visa security group, indicating that those who changed from low to high visa security reported significantly greater decreases in depression symptoms compared to those who maintained low visa security (see Figure 1). Furthermore, there was a significant interaction between time and remaining in the high visa security group, indicating that those who maintained high visa security reported significantly greater decreases in depression symptoms over time compared to those who maintained low visa security. There was no significant interaction between time and remaining in the medium visa security group or changing from low to medium visa security, indicating that there was no difference in change in depression symptoms between those who retained medium visa security or transitioned from the low to medium visa security groups and those who maintained low visa security.

For immigration-related fears, there was a significant interaction between time and changing from the low to high visa security group, indicating that participants who changed from low to high visa security reported a greater reduction in immigration-related fears than those who maintained low visa security. There was also a trending to significant interaction between time and changing from the low to medium visa security group (at *p* = 0.50), indicating that participants who changed from low to medium visa security reported a greater reduction in immigration-related fears than those who maintained low visa security (see Figure 1). There was no significant interaction between time and remaining in the high visa security group or remaining in the medium security group, indicating that there was no significant difference in change in immigration-related fears between those who maintained high, medium or low visa security.

For social integration difficulties, there was a significant interaction between time and change between the low to high visa security group, indicating that those who changed from low to high visa security reported significantly greater decreases in social difficulties compared to those maintained low visa security (see Figure 1). Furthermore, there was a significant interaction between time and remaining in the high visa security group, indicating that those who maintained high visa security reported significantly greater decreases in social difficulties over time compared to those who maintained low visa security. There was no significant interaction between time and changing from low to medium visa security, or between time and maintaining medium visa security group indicating that there was no significant difference in change in social difficulties between those who changed from the low to medium visa security groups or maintained medium visa security and those who maintained low visa security.

## Discussion

To our knowledge this is the first study to investigate whether changing from low visa security (e.g. no visas, expired visas, and bridging visas) to medium visa security (e.g. temporary protection visas) led to improved psychological and social outcomes among refugees. The finding that refugees who changed from low visa security to high visa security showed significant decreases in depression symptoms is consistent with research that has demonstrated the positive association between permanent visa status and mental health among refugees (Momartin et al., 2006; Newnham et al., 2019; Nickerson et al., 2019; Steel et al., 2006, 2011). This finding is also in accordance with a previous study that found that moving from medium security (temporary protection) visas to high security (permanent protection visas) was associated with significant reductions in depression symptoms, extending these findings to refugees who moved from low to high security (Nickerson et al., 2011). In contrast, refugees in the current study who moved from low to medium security visas showed similar patterns of depression symptoms over time to those who maintained low visa security, as did those who maintained medium visa security. In the Australian context, until recently refugees with temporary protection visas were not eligible for permanent protection, were obliged to periodically re-apply for temporary protection (and demonstrate their claim for refugee protection), often experienced substantial delays (e.g. several years) in processing of visa status, and had limited access to legal and language assistance (Kenny et al., 2022). Results from this study suggest that remaining in a state of prolonged insecurity (compared to experiencing increased security afforded by permanent protection) may be associated with poorer long-term mental health outcomes (Kenny et al., 2022; Law, 2022b). This is consistent with a broader body of research highlighting the importance of a safe, controllable and predictable environment to facilitate mental health recovery following exposure to adversity (Foa et al., 1992; Frazier et al., 2004).

We observed a similar pattern for social integration difficulties between groups: refugees who moved from low to high visa security demonstrated significant improvement in social integration problems compared to those who maintained low visa security, which wasn’t observed for those that moved from low to medium security visas. This finding extends previous research which has predominantly focused on mental health implications of visa security (Nickerson et al., 2011), to suggest that the provision of permanent, but not temporary protection, may also facilitate positive social adaptation. One factor that may contribute to poorer social adaptation is the fact that, in Australia, until recently, refugees with temporary protection had no access to family reunification (Law, 2022b). Research has consistently documented the negative psychological and social impact of family separation in refugees (Liddell et al., 2021; Miller et al., 2018; Rousseau et al., 2001). Furthermore, when family members remain in dangerous contexts where they are subject to war and/or persecution, the inability to seek family reunification may substantially exacerbate distress in TPV holders, and remove critical pathways to safety for family members (Liddell et al., 2022). These ongoing stressors and uncertainties may be a contributing factor that could hamper the capacity of refugees to adapt to the host society.

The finding in this study that participants who moved from low to medium security visas showed trends of greater decreases in immigration-related fears than those who maintained low visa security is notable as it suggests that the provision of temporary protection may have reduced fears of immediate deportation. Taken together, however, our findings suggest that the level of short-term security afforded by temporary protection policies (as evidenced by decreases in immigration-related fears) may not be adequate to support improvement in psychosocial functioning for refugees who have been exposed to significant persecution and conflict-related trauma in their home countries.

Study findings should be interpreted in the context of limitations. First, the number of refugees who transitioned from low to high security in this study was small. This is reflective of the Australian system where this pathway was uncommon at the time of data collection (2015–2020). While we found a large change in outcomes for this group (suggesting there was adequate power), this group may not be broadly representative of refugees with temporary visas. Second, we cannot rule out pre-existing differences between visa security groups contributing to findings. The group who transitioned from low to medium security visa predominantly arrived by boat, likely without a valid visa, and were informed that they would never permanently live in Australia. In contrast, those who transitioned from low to high security visas in this study arrived predominantly by plane with a valid visa, and likely had an expectation of being provided with high security visas. This is reflected in the finding that those in the former group had higher immigration-related fears at the outset of the study than those in the latter group. Nevertheless, the fact that these two groups did not differ in depression symptoms or social difficulties at baseline and that there was significant improvement in both of these after receiving high security visas indicates that, over and above these potential differences, the provision of high security visas was associated with improved wellbeing and functioning. Third, this study implemented a largely online data collection method. While this has benefits in terms of privacy and removal of demand characteristics, it may be that interview-based methods could have gathered more accurate estimates of study outcomes, as well as more detailed visa information. Furthermore, participants with relatively low literacy or digital literacy may have been excluded in this study, possibly limiting the generalizability of the findings to those with higher education and/or younger age. Fourth, this study implemented convenience sampling rather than representative sampling, meaning that the findings may not be generalizable to all refugees. Finally, our measures of immigration-related fears and social difficulties were relatively brief and could be more robustly measured by more extensive batteries in future studies.

Findings from this study have substantial implications for policy and practice. In the context of unprecedented levels of forced displacement, many countries are seeking new solutions to managing the influx of refugees, including temporary protection. This study suggests that the positive psychological and social effects afforded by permanent protection following displacement due to conflict and persecution may not extend to temporary protection, apart from alleviating immediate immigration-related fears. Indeed, the United Nations High Commissioner for Refugees asserts that policies that implement forms of insecure visas are not in line with international commitments to refugee protection, specifying that temporary protection should not be considered a replacement for permanent protection when the latter is available, nor should it be implemented for prolonged stays (UNHCR, 2014). The current findings provide support for the restriction of temporary protection to brief crisis periods (UNHCR, 2014) (such as has been recently implemented by several countries in the context of providing immediate protection to people displaced by the war in Ukraine), and suggest that the rapid provision of permanent protection is likely to lead to better psychological and social outcomes for refugees. This indicates that increasing the number of permanent resettlement places instead of adopting temporary protection policies may have positive consequences for refugees’ wellbeing, as well as their capacity to adapt socially to the host environment. Following this, the Australian Government recently announced a plan to convert all TPVs who arrived in Australia prior to the 14 February 2023 to permanent visas via the Resolution of Status pathway (Australian Government Department of Home Affairs, 2023). This action moves towards providing refugees living in a state of protracted insecurity with greater certainty and control over their future. Nevertheless, individuals who arrive in Australia without a valid visa after this date, and are subsequently found to be refugees, may not be eligible for permanent or high security visas, and thus may be subject to the insecurity associated with temporary protection. Findings also suggest that targeted mental health services and enhanced opportunities for social connection and engagement (including access to family reunion pathways) are potentially important strategies to support refugees living with prolonged insecurity.

This study demonstrated that the provision of permanent visas was associated with significant improvements in mental health, immigration-related fears and social integration difficulties among refugees in Australia who previously held visas with low levels of security. In contrast, the provision of temporary visas was only associated with improvements in immigration-related fears. These findings highlight the potentially significant mental health and social burden of living in a prolonged state of insecurity. Study results provide preliminary evidence for targeted service provision for refugees with low visa security, as well as policy change to facilitate positive psychological and social adaptation.

## Notes

1. In Australia, Bridging Visas (classified as low security visas in this study) are temporary visas that allow individuals to reside in Australia for a specified period while their substantive visa application (e.g. claim for refugee protection) is processed (Andrew & Renata Kaldor Centre for International Refugee Law, 2000a). Asylum-seekers who arrived in Australia by plane or boat without a valid visa, or those who have previously been in immigration detention, were provided with a particular type of Bridging Visa (Bridging Visa E). Conditions of these visas restricted many individuals from receiving social security benefits and/or working in Australia. Temporary protection visas (classified as medium security visas in this study) are provided to individuals who had arrived in Australia without a valid visa, and whose claim to refugee status has subsequently been recognized (UNSW Kaldor Centre for International Refugee Law, 2000b). Accordingly, individuals on Bridging Visas may be subsequently eligible for either TPVs or permanent visas. Until February 2023, refugees with TPVs were ineligible to apply for permanent protection, instead having to re-apply for TPVs every 3 or 5 years. Australia recently changed its policy relating to TPVs, however, and has introduced pathways by which those who received TPV status prior to a certain date can now apply for permanent protection visas (Australian Government Department of Home Affairs, 2023). As this policy was changed after data collection, it does not impact on findings presented in this study.
